# Relationship between bull signalment and testicular attributes in a Kenyan bull station

**DOI:** 10.14202/vetworld.2024.2072-2076

**Published:** 2024-09-15

**Authors:** Peterkin Nzomo Munywoki, Ambrose Ng’eno Kipyegon, Wilkister Nakami Nabulindo, Roselyne Wambugu, David Kios

**Affiliations:** 1 Department of Clinical Studies, Faculty of Veterinary Medicine, The University of Nairobi, Nairobi, Kenya; 2 Kenya Animal Genetic Resources Center, Nairobi, Kenya

**Keywords:** age, body weight, breed, scrotal circumference, testicular ultrasonography

## Abstract

**Background and Aim:**

Male fertility is essential to bovine reproduction, particularly when bulls are used for artificial insemination or single-sire breeding. Bull breeding and soundness examinations (BBSE) are routinely undertaken to identify potentially unfit bulls for breeding. Multiple criteria, including physical examination and determination of testicular and semen parameters, characterize BBSE. Knowledge interstices within this realm, especially in tropical African settings, necessitate pragmatic approaches to address the same. This study aimed to investigate the potential effects of bull parameters on testicular attributes in a tropical setting.

**Materials and Methods:**

The present study recruited healthy bulls (n *=* 96) aged >16 months with active semen collection used for artificial insemination at the Kenya Animal Genetic Resources Center. The breed breakdown was as follows: Ayrshire (n *=* 40), Boran (n *=* 3), Friesian (n *=* 36), Guernsey (n *=* 5), Jersey (n *=* 7), and Sahiwal (n *=* 5). Age, breed, body weight, scrotal circumference (SC), and testicular echotexture were collected, and the findings were analyzed using R statistical software.

**Results:**

SC increased with age and body weight (p < 0.0001). SC varied from one breed to the others (p < 0.0001). Furthermore, as determined using trans-scrotal ultrasonography, hyperechoic testicular lesions were present in 30.21% of the bulls imaged, and the incidence was significantly related to age (p < 0.001).

**Conclusion:**

SC is significantly affected by age and body weight. The mean SC was higher in the *Bos indicus*, but this finding is only indicative because the Sahiwal and Boran sample sizes were small. Notably, the prevalence of hyperechoic testicular foci following trans-scrotal ultrasonography was common in older bulls. However, there is a need to further elucidate this phenomenon’s pathophysiology with age as the etiology and possible sequelae of semen quality.

## Introduction

Male fertility is essential for bovine reproduction because individual bulls serve numerous cows [1]. Since the discovery and use of artificial insemination (AI) in the 1950s, bull breeding and soundness examination (BBSE) have been routinely undertaken to identify bulls that are potentially unfit for breeding [2]. Approximately 20%–40% of the bulls screened fail their BBSE and are deemed infertile. However, there are very few truly sterile bulls [3]. Multiple sire groups used for breeding may mask bulls with sub-fertility, but AI and single-sire groups emphasize the need to evaluate bull fertility [3]. No single criterion or parameter reliably predicts bull fertility, thereby sanctioning the use of numerous criteria. The classification involves physical examination in which bulls must meet specific testicular development thresholds, sperm motility, and morphology. Although bulls should be free from venereal diseases and exhibit normal libido, they are not routinely evaluated during BBSE [3]. BBSE includes assessing body size and condition, muscling, scrotal circumference (SC), and freedom from physical defects and diseases [4].

Bulls with a large SC produce daughters and half-sib heifers with high fertility and early puberty [4]. Previous studies have revealed that semen quality is affected by numerous factors, including but not limited to age, season, SC, breed, and body weight [5–9]. SC is highly correlated with paired testicular weight, testosterone, and semen quality [4, 10, 11] and is expected to be >31 cm at 15 months of age [12]. Calves with an SC >23 cm on 200 days have a 95% chance of attaining an SC >34 cm at 12 months [13]. Other studies have reported that SC increases with body weight [9, 14, 15]. Trans-scrotal ultrasonography is a noninvasive tool for identifying testicular and epididymal lesions [16, 17]. Testicular fibrosis has been observed in bulls as early as 5–6 months with unclear pathology because spermatogenesis starts at 8–10 months [18]. However, literature comparing pixel intensity (PI) and its effect on semen quality is scarce [16]. Furthermore, studies that have compared the two variables have revealed an unclear impact of PI on semen quality [19] or no relationship [20].

This study compared bull signalment (age, body weight, and breed) with testicular attributes (SC and testicular echotexture) in bulls used for AI at the Kenya Animal Genetics Resource Center (KAGRC). The study area is in a tropical setting, where indigenous breeds remain largely understudied. Coupled with the breed diversity and the different adaptations of *Bos taurus* to different ecological zones, a study was necessary. Furthermore, to the best of our knowledge, the present study is the first in Kenya, thus providing invaluable insight into sub-Saharan Africa’s livestock fertility and bull selection criteria.

## Materials and Methods

### Ethical approval

Ethical approval was obtained from the BACU Faculty of Veterinary Medicine (REF: FVM BAUEC/2023/418).

### Study period and location

The study was conducted from January 2022 to March 2022 at The Kenya Animal Genetic Resource Center at −1.24238767621 (Latitude), 36.7338180542 (Longitude) Lower Kabete, Nairobi, Kenya, at 1777.36 m above sea level. The study recruited bulls of different breeds (n *=* 96), and the breed breakdown was as follows: Ayrshire (n *=* 40), Boran (n *=* 3), Friesian (n *=* 36), Guernsey (n *=* 5), Jersey (n *=* 7), and Sahiwal (n *=* 5). The bulls met the inclusion criteria (above 16 months, healthy, and in active semen collection).

### Determination of bull signalment and testicular attributes

The study was cross-sectional in nature; individual bull records were used to determine breed and date of birth. Body weight was determined using a livestock weighing scale (AEA LTD, Nairobi, Kenya). The bulls were then restrained in a crush, and SC was measured using scrotal tape (Lane Manufacturing Co., Denver, CO, USA) as described by Foote [21].

A portable ultrasound machine (Minitüb GmbH, Hauptstraße 41, 84184 Tiefenbach, Germany) was used for trans-scrotal ultrasonography to determine the testicular echotexture. The testes were pushed ventrally and retained, and a coupling gel was uniformly applied to the footprint. B-mode ultrasonography using a linear probe at a frequency of 6.5 MHz was performed to image the testes, and both the cranial and caudal surfaces of the testes were imaged using a sagittal view. The testicular ultrasonograms were collectively analyzed and interpreted subjectively, and the findings were deemed normal or abnormal depending on the presence of hyperechoic testicular lesions.

### Statistical analysis

Data were recorded and exported to Microsoft Excel 2016 (Microsoft Corporation; Redmond, WA) and R statistical software v. 2023.06.0+421 [22] for analysis. Simple linear regression models were used to determine the effect of age and body weight (predictors) on SC (criterion). The normality of the data was determined using a Shapiro-Wilk test, and parametric tests were indicated for data with a normal distribution. Fisher’s one-way analysis of variance (ANOVA) determined SC variance in one breed from the next.

Logistic regression analysis was used to determine the relationship between age and the prevalence of hyperechoic testicular lesions. Differences were considered statistically significant when p < 0.05. Fisher’s one-way ANOVA followed a Shapiro-Wilk normality test (p > 0.05) and Levene’s test.

## Results

### Relationship between age and SC

**Figure-1:**
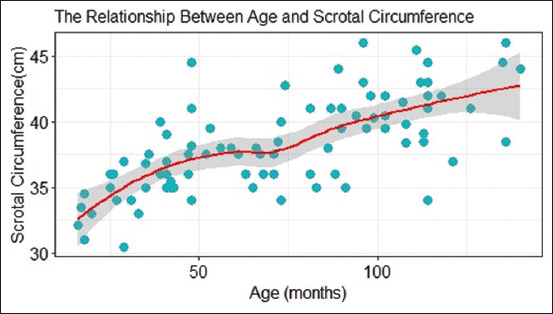
Scatter plot with a geom line showing the positive effect of age on scrotal circumference.

The bulls were classified into five different age groups, and their respective SC means are presented in Table-1. A linear regression analysis showed that age significantly affected SC (p < 0.0001). Bulls below 3 years of age had the lowest SC (34.41 cm), whereas the SC was highest in bulls above 9 years (40.33 cm). A scatterplot in Figure-1 illustrates this finding.

**Table-1:** Bull mean SC across different age groups.

Age group (years)	Number of bulls (n)	SC Mean
≤3	18	34.41 ± 1.994961
>3–5	20	37.39 ± 2.541503
>5–7	18	37.29 ± 2.266501
>7–9	21	40.33 ± 2.450578
>9	19	41.53 ± 3.061781

### Relationship between body weight and SC

**Figure-2:**
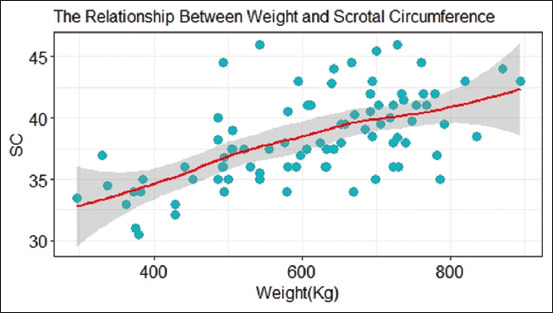
Scatter plot with a geom line showing the positive effect of weight on scrotal circumference.

A linear regression analysis revealed a significant relationship between body weight and SC (p < 0.0001). The SC was lowest in bulls weighing 400 kg and highest in bulls weighing >800 kg (Figure-2).

### Effect of breed on SC

**Figure-3:**
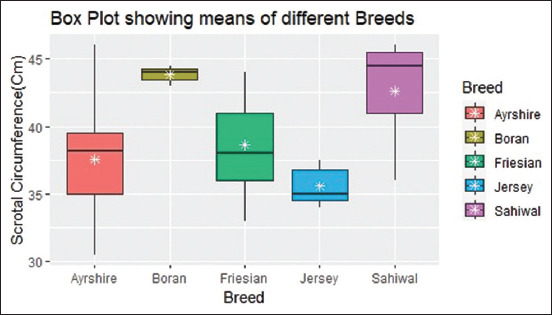
Box plot showing the 5-number summary and mean scrotal circumference of different breeds.

SC varied significantly from one breed to the next, as indicated by the p < 0.0001, F statistic (5.80), and an ES value 0.20 (>0.14) at 95% confidence level (CI) [0.06, 1.00]. The Bayes factor indicated that breed had a powerful effect on SC (−4.29), and the R^2^ value (0.18) at 95% CI [0.04, 0.32] indicated that the model moderately favored the data. SC was highest in the *Bos indicus* and lowest in the *B. taurus* (Figure-3).

### Effect of age on testicular echotexture

**Figure-4:**
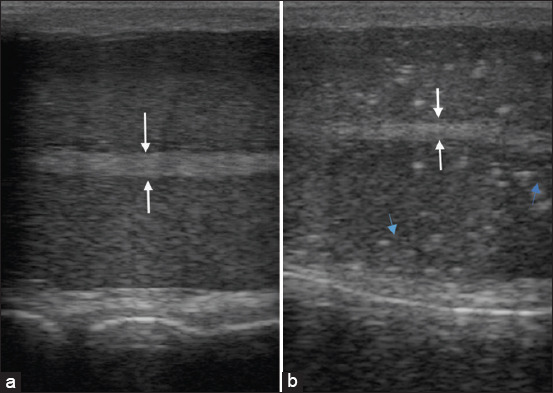
(a) Normal testes with relatively homogenous parenchyma and stippled medium echogenicity. The parenchyma surrounds a centrally located and more hyperechoic mediastinum testis (white arrows). (b) Small scattered hyperechoic microfoci (blue arrows) are randomly distributed in the parenchyma, which is characteristic of hyperechoic testicular lesions.

Testicular ultrasound examination revealed that 30.21% (n = 29) had hyperechoic testicular microfoci, whereas 69.79% (n = 67) had normal findings. The results in Figure-4a were considered normal, whereas those in Figure-4b were considered abnormal after the observation of hyperechoic microfoci. Logistic regression revealed that age was significantly related to this phenomenon (p < 0.001). All 36-month-old bulls had normal testicular ultrasound findings. Hyperechoic testicular lesions were first noticed at 48 months, and the prevalence was highest in bulls above 84 months (62.06%).

The white arrows in Figures-4a and b show the mediastinum testis, whereas the blue arrows in Figure 4b show hyperechoic microfoci, which are indicative but not confirmatory of age-related fibrosis. The scrotal walls appear ventrally and dorsally. The testicular parenchyma is hypoechoic.

## Discussion

SC is an essential parameter in breeding and soundness examination. A large SC is desirable because it is correlated with good semen quality [4, 11]. Bulls aged >12 months should have an SC of >31 cm [23]. In the present study, the youngest bull (16 months) had an SC of 32 cm, and the oldest bull (140 months) had an SC of 44 cm.

Bulls aged below 36 months had the lowest SC (34.41 cm) and the largest SC was recorded in bulls aged >108 months (41.53 cm) (p < 0.0001). These findings are consistent with those of previous reports by Brinks [24], Perumal [25], and Brito *et al*. [26]. The present study showed that SC increased exponentially up to 60 months. Beyond 60 months, SC increases but at a relatively slower rate, even in bulls aged >100 months, contrary to a previous study by Ahmad *et al*. [27], who reported that SC growth plateaus beyond 100 months.

Body weight affects SC, with heavy bulls exhibiting a large SC [9]. The present study reported a significant relationship between SC and bull weight (p < 0.0001), and these findings are consistent with those of previous studies by Menegassi *et al*. [9], Waite *et al*. [14], and Indriastuti *et al*. [15]. SC varies significantly between breeds in *B. indicus* and *B. taurus* (p < 0.0001) across all ages. Unlike Brito *et al*. [26], who found no significant effects of breed on SC in *B. indicus*, *B. taurus*, and crossbreeds, the present study reports significant variation among *B. indicus* and *B. taurus*, where *B. indicus* had a larger SC. Individual significant variations in SC were also reported among *B. taurus*.

Boran had the largest SC (43.83 cm), while Jersey had the lowest SC (35.57 cm). The Sahiwal had the second-largest SC (42.60 cm), higher than a similar study by Ahmad and Asmat [28], who used Sahiwal of similar age (>5 years) and reported a mean SC of 32.38 cm. The comparison between the *B. indicus* and the *B. taurus* is not conclusive but indicative due to the small *B. indicus* sample size (n *=* 8). Literature comparing SC across different breeds, however, remains scarce.

Ultrasound findings revealed hyperechoic testicular microfoci in 30.21% (n = 29) of the bulls. The sagittal view revealed excellent exposure of the mediastinum testes, dorsal and ventral scrotal borders, and testicular parenchyma on both caudal and cranial views. The onset of the observed testicular lesions was primarily influenced by age, as revealed by a logistic model (p < 0.001). A similar study reported that testicular fibrosis with an unclear etiology occurs as early as 5–6 months, even before spermatogenesis [18].

Degenerative testicular changes have a multifactorial etiology, including disease, trauma, environmental factors, and age-related idiopathic changes [29]. Tissue cysts from bulls chronically infected with *Besnoitia besnoiti* have been identified in the scrotal skin pampiniform plexus and testicular parenchyma [30]. Bulls with severe besnoitiosis develop early sterility with marked inflammatory response and vascular injury [31]. However, in the present study, abnormal testicular echotexture was not reported until 48 months. Most cases (62.06%) were recorded in bulls aged >84 months, indicating age as a potential precipitant of hyperechoic testicular lesions.

## Conclusion

SC increases with age and body weight and significantly varies from breed to breed. *B. indicus* (Sahiwal and Boran) has a larger SC than *B. taurus* (Ayrshire, Friesian, Guernsey, and Jersey). This finding is, however, indeterminate but indicative given the small *B. indicus* sample size, and future studies can build on it by including a larger sample size. Finally, there is an increasing trend in the prevalence of hyperechoic testicular microfoci with increasing age. However, further research is needed to understand the effects of this phenomenon on semen quality.

## Authors’ Contributions

PNM, ANK, and WNN: Conceptualized and designed the study. PNM: Prepared and revised the manuscript. ANK and WNM: Supervised data collection and manuscript preparation and editing. RW and DK: Facilitated data collection at KAGRC. All authors have read, reviewed, and approved the final manuscript.
